# Fallbericht: Tod eines 2-jährigen Mädchens bei postmortaler Diagnose einer seltenen, Kawasaki-Syndrom-typischen Koronararterienvaskulitis

**DOI:** 10.1007/s00194-021-00482-9

**Published:** 2021-04-23

**Authors:** K. Kanngießer, N. Kono, J.-T. Suhren, M. Klintschar

**Affiliations:** 1 Institut für Rechtsmedizin, Medizinische Hochschule Hannover, Carl-Neuberg-Str. 1, 30625 Hannover, Deutschland; 2 Institut für Pathologie, Medizinische Hochschule Hannover, Carl-Neuberg-Str. 1, 30625 Hannover, Deutschland

**Keywords:** Plötzlicher Tod, Kawasaki-Syndrom, Behandlungsfehler, Koronararterienaneurysma, Myokardinfarkt als Kleinkind, Sudden death, Kawasaki syndrome, Medical malpractice, Aneurysms of the coronary arteries, Myocardial infarction in a toddler

## Abstract

Das Kawasaki-Syndrom, eine Autoimmunvaskulitis der Koronararterien, ist eine zumindest in Deutschland seltene Erkrankung, welche sich typischerweise im Kindesalter manifestiert. Die Symptomatik ist weitgehend unspezifisch, wobei die Erkrankung zu schwerwiegenden Komplikationen mit Ausbildung von Gefäßaneurysmen und Thrombosen bis hin zum Herzinfarkt führen kann. Im Zusammenhang mit der COVID-19-Pandemie sind seit Anfang letzten Jahres gehäuft Fälle mit Kawasaki-Syndrom-ähnlichem Erkrankungsbild bei Kindern mit positivem SARS-CoV-2-Nachweis bekannt geworden.

Aus aktuellem Anlass wird daher über ein 2 Jahre altes Kind aus der Prä-COVID-19-Ära berichtet, welches über 6 Tage an Fieber litt. Nach vorübergehender Besserung entwickelte sich dann innerhalb eines Tages eine akute und letztlich tödlich verlaufende Verschlechterung des Gesundheitszustandes.

Im Rahmen der Obduktion und weiterführender Untersuchungen konnte eine Autoimmunvaskulitis der rechten und des Hauptstamms der linken Koronararterie mit Aneurysmabildung, vereinbar mit einem Kawasaki-Syndrom, nachgewiesen und als Todesursache ein Herzinfarkt auf dem Boden thrombotischer Verschlüsse der Koronararterienaneursymen festgestellt werden.

Gerade vor dem Hintergrund der aktuellen Ereignisse sollte bei fieberhaften sowie viralen Infekten in der Vorgeschichte und autoptisch makroskopisch auffällig dilatierten bzw. thrombosierten Koronararterien bzw. Herzinfarkten im Kindesalter neben anderen Differenzialdiagnosen auch ein Kawasaki-Syndrom ausgeschlossen werden.

## Abstract (en)

Coronary artery autoimmune vasculitis (Kawasaki syndrome) is at least in Germany a very rare condition, that typically manifests in childhood. The symptoms are often unspecific and complications with vascular aneurysms, thrombosis and myocardial infarction can occur. Multiple cases of Kawasaki-like symptoms in children with positive SARS-CoV‑2 test results have been reported during the course of the COVID-19 pandemic the past year.

This case study reports on a 2-year-old child who had fever over 6 days and after a temporary improvement, died within 1 day (pre-COVID19 era).

The autopsy showed autoimmune vasculitis of the right and left main coronary artery consistent with Kawasaki syndrome with aneurysm formation, acute thrombosis and myocardial infarction.

In the case of macroscopically conspicuous dilated and/or thrombosed coronary arteries and/or myocardial infarction in children, a Kawasaki syndrome should be excluded in addition to other differential diagnoses.

## Einleitung

Koronarvaskulitiden im Kindesalter sind selten und schwer zu diagnostizieren [1]. Sie kommen u. a. im Rahmen des Kawasaki-Syndroms vor [1] und gehen dann häufig mit Symptomen wie einer schmerzhaften Lymphknotenschwellung, Haut‑/Schleimhautveränderungen sowie Fieber einher, weshalb es auch als mukokutanes Lymphknotensyndrom bezeichnet wird [8, 9]. Obwohl die Erkrankung in Europa mit einer Inzidenz von 5/10.000 selten vorkommt [12], gilt die autoimmune Vaskulitis der Koronararterien mit Ausbildung von Aneurysmen als schwerwiegende und gefürchtete Komplikation [3–5, 7, 11, 13, 16] und zählt zu den führenden Ursachen erworbener Herzerkrankungen im Kindesalter [11]. Die Morbidität und Mortalität der Erkrankung werden hierbei maßgeblich durch das Auftreten koronararterieller Veränderungen beeinflusst [3–5, 11]. Prinzipiell kann die Prävalenz dieser tödlichen Komplikationen bei zeitnaher Diagnosestellung und Therapie deutlich gesenkt werden [5]. Erstere erfolgt anhand definierter Kriterien (Tab. 1), wobei Fieber, welches im Kindesalter jedoch ein häufiges und unspezifisches Symptom bildet, als Leitsymptom gilt [3–5, 7, 11, 13]. Sowohl das unspezifische klinische Erscheinungsbild als auch die von vielen Autoren als zu spezifisch angemahnten Diagnosekriterien [3, 5, 15] führen in der Praxis häufig zu einer verzögerten Diagnosestellung und Therapie mit nicht selten tödlichem Ausgang.

**Table 1:** 

*1.*	*Persistierendes Fieber seit mindestens 5 Tagen*	
*2.*	*Mindestens 4 der folgenden 5 Hauptsymptome*	
	a.	Extremitätenveränderungen (akut Erytheme und Ödeme an Händen und Füßen, im Verlauf Hautablösungen im Bereich der Fingerspitzen)
	b.	Polymorphes Exanthem
	c.	Bilaterale, schmerzlose konjunktivale Injektion ohne Exsudat
	d.	Veränderungen im Bereich der Lippen und der Mundhöhle (Erythem und Rissigkeit der Lippen, Erdbeerzunge, diffuse Injektion der oralen und pharyngealen Schleimhäute)
	e.	Zumeist unilaterale zervikale Lymphadenopathie (Durchmesser >1,5 cm)

Besondere Aktualität verleiht dieser an sich sehr seltenen Erkrankung, dass im Zuge der weltweiten Ausbreitung des SARS-CoV‑2 bis Ende April 2020 insbesondere in Europa rund 100 Fälle von Kindern im Alter zwischen 6 Monaten und 17 Jahren mit einem dem Kawasaki-Syndrom ähnlichen Erkrankungsbild im Zusammenhang mit einer COVID-19-Erkrankung berichtet wurden; bis Anfang Mai 2020 verliefen dabei insgesamt 4 Fälle tödlich [12].

Dieser Bericht eines (allerdings nicht mit COVID-19 assoziierten bzw. aus der Prä-COVID19-Ära) Falles soll verdeutlichen, wann im Rahmen einer rechtsmedizinischen Obduktion an ein Kawasaki-Syndrom gedacht werden sollte.

## Fallbericht

### Vorgeschichte

Ein knapp 2‑jähriges, bisher laut Angaben der Eltern gesundes Mädchen litt seit 6 Tagen an Fieber, welches sich unter nicht näher bekannter antipyretischer Therapie zunächst besserte; eine ärztliche Vorstellung erfolgte deshalb nicht. Am Tag des Todes kam es jedoch zu einer akuten Verschlechterung des Allgemeinzustandes, und das Kind verstarb trotz notfallmedizinischer Maßnahmen.

### Makroskopische Obduktionsbefunde

**Figure 1:**
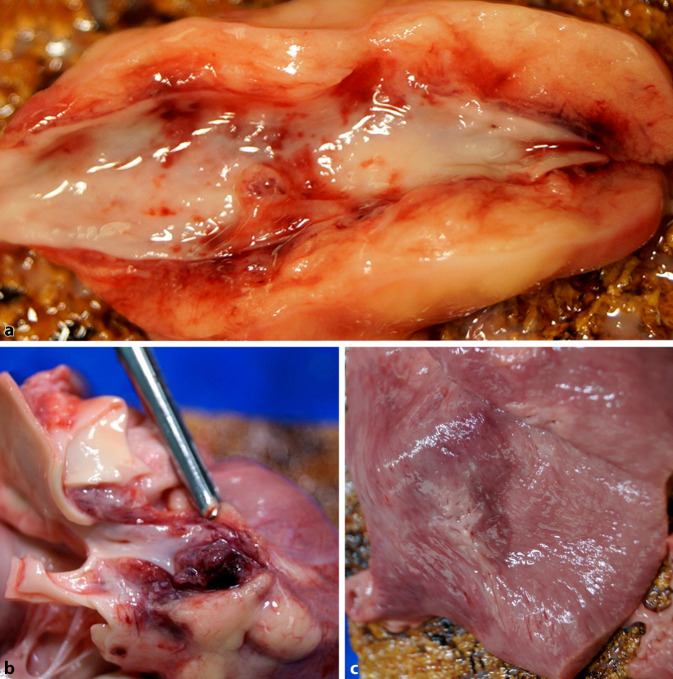

Bei der Obduktion zeigte der Leichnam einen altersentsprechenden Entwicklungs- und Ernährungszustand (Körpergewicht 13 kg, Köpergröße 93 cm). Bei altersgemäßem Herzgewicht (90 g) und regelhaftem Vier-Kammer-Aufbau des Herzens fielen epikardial kleinfleckige Einblutungen und ein seröser Perikarderguss von 40 ml auf. Die Hauptstämme beider Koronararterien wiesen zudem jeweils abgangsnah etwa 3 cm lange, aneurysmatische Ausweitungen von bis zu 1,5 cm Umfang auf (Abb. 1a, b); die Gefäßlumina waren hier jeweils durch scheinbar festsitzende, dennoch frisch imponierende Thromben weitgehend verlegt (Abb. 1a, b), wobei im Bereich der kräftig aufgerauten Intima fleckförmige Einblutungen auffielen. In der linken Herzseiten- und Hinterwand war eine bis zu 5 cm durchmessende Infarktabblassung abgrenzbar (Abb. 1c). Darüber hinaus ließen sich ein massives Hirnödem und ausschließlich flüssiges Leichenblut feststellen. Trachea, Bronchien und Milz wiesen allenfalls gering entzündliche Veränderungen auf. Ferner zeigten sich, neben subpleuralen Einblutungen und beidseitigen Pleuraergüssen, blutreiche und luftarme Lungen.

### Mikroskopie und weitere Zusatzuntersuchungen

**Figure 2:**
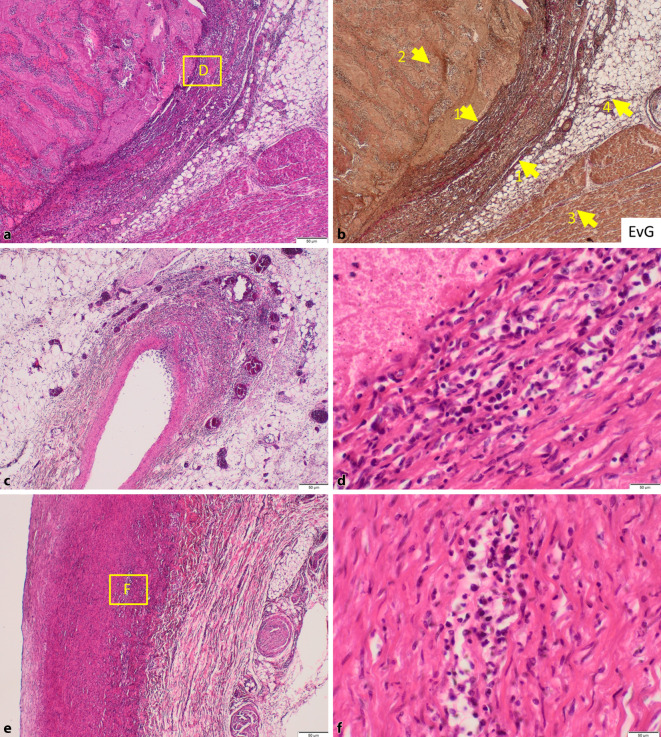

Mikroskopisch fand sich eine autoimmunvaskulitistypische segmentale lymphohistiozytäre Entzündung der Gefäßwände der Koronararterien mit teils fibrinoiden Nekrosen der Media sowie luminal frischen fibrinreichen Thrombusanteilen (Abb. 2a–d). Mikroskopisch zeigte sich keine Organisation des Thrombus, sodass die makroskopisch beschriebene, vermeintliche Wandhaftung der schweren Endothelschädigung zuzuschreiben ist. Immunhistochemisch zeigte sich ein Entzündungsinfiltrat aus Histiozyten (CD68), gemischt CD4+ und CD8+ T-Lymphozyten mit partieller Koexpression von TIA (keine Vermehrung von Granzym B oder Perforin) sowie sehr wenigen B‑Zellen und Plasmazellen (CD20, CD138). Zudem ließ sich mikroskopisch eine geringe lymphozytäre Aortitis nachweisen, wobei keine Granulome, keine eitrige Entzündung, Pilzbesiedlung oder Aortensklerose vorlagen (Abb. 2e, f).

Virale Infektionen ließen sich nicht nachweisen (Influenza A und B, Adeno‑, Metapneumo‑, Picorna‑, Rhino- und Enterovirus).

Die chemisch-toxikologische Analyse von Herzblut und Mageninhalt auf gängige Arznei- und Betäubungsmittel verlief negativ.

### Obduktionsdiagnose

Als Todesursache konnte eine Autoimmunvaskulitis beider Koronararterien mit aneurysmatischen Aussackungen, vereinbar mit einem Kawasaki-Syndrom, und Ausbildung eines akuten Herzinfarktes auf dem Boden einer thrombotischen Verlegung der Koronararterien festgestellt werden.

## Diskussion

Das Kawasaki-Syndrom bezeichnet eine in der Regel im Kindesalter auftretende, selbstlimitierende, generalisierte Vaskulitis der kleinen und mittleren Gefäße mit Prädilektionsstelle im Bereich der Koronararterien [3–5, 11]. Wenngleich die genaue Ursache bislang nicht hinreichend geklärt ist, scheint die Aktivierung des Immunsystems durch einen noch unbekannten Stimulus eine zentrale Rolle zu spielen [11]. In der Folge kommt es zu einer durch T‑Zellen (insbesondere CD8-positive zytotoxische T‑Zellen) und Makrophagen vermittelten Vaskulitis sowie einer vermehrten vaskulären Permeabilität mit Thrombozytenaggregation und Sekretion proinflammatorischer Zytokine [4]. Die für das Kawasaki-Syndrom typischen klinischen Zeichen mit Fieber, Haut‑/Schleimhautveränderungen und Lymphadenopathie dürften dabei einer systemischen Mitreaktion entsprechen [4]. Die Morbidität und Mortalität der Erkrankung werden maßgeblich durch das Auftreten von Koronararterienschädigungen in der akuten Krankheitsphase beeinflusst [3–5, 11]. Bei etwa 8 % der Patienten kommt es zur Ausbildung koronararterieller Aneurysmen [11], und bei etwa 0,5–2 % aller Patienten verlaufen die Komplikationen tödlich [3]. Darüber hinaus können auch Jahre später noch Komplikationen auftreten, wobei etwa 5 % der akuten Koronarsyndrome bei Erwachsenen unter 40 Jahren auf Kawasaki-Syndrom-assoziierte koronare Aneurysmen zurückzuführen sind [11]. Die Aneurysmabildung erklärt sich dadurch, dass es im Rahmen der Gefäßentzündung zu einer myofibroblastären Proliferation mit Verdickung der Intima und gleichzeitig zu einer Ausdünnung und Fibrose der Media kommt [3, 4, 11]. Die Abgrenzung zu anderen Ursachen für einen plötzlichen Herztod ist schwierig und bedarf meist der histologischen Aufarbeitung [6, 10]. Eine klare Differenzierung zur infantilen Polyarteriitis ist dabei nicht möglich [1, 2, 14]. Vielmehr weisen beide Krankheitsbilder sowohl klinisch als auch makro- und mikroskopisch nahezu gleichartige Charakteristika auf, welche zumindest teilweise auch im vorgestellten Fallbeispiel vorzufinden waren (Tab. 2).

**Table 2:** 

	Kawasaki Syndrom	Polyarteriitis nodosa	Vorgestellter Fall
*Betroffene Gefäße*	Koronararterien	Mittelgroße Gefäße (unter anderem auch Koronararterien möglich)	Koronararterien und fokal Aorta
*(Durchschnittliches) Alter (Jahre) bei Krankheitsbeginn*	<5	7–12	2
*Histopathologie*	Lymphohistiozytäre Vaskulitis mit fibrinoider Nekrose der Arterienwand	Lymphohistiozytäre Vaskulitis mit fibrinoider Nekrose der Arterienwand	Lymphohistiozytäre Vaskulitis mit fibrinoider Nekrose der Arterienwand sowie als Komplikation ein frischer Thrombus
*Klinische Symptomatik*	Fieber, Konjunktivitis, Ausschlag, Lymphadenopathie, Erythem des Oropharynx, der Handflächen und Fußsohlen	Fieber, Myalgie, Arthralgie, Livedo reticularis, subkutane Knoten, Nieren- und Nervenbeteiligung	Fieber (klinische Angaben lückenhaft)

Bei frühzeitiger Diagnosestellung eines Kawasaki Syndroms innerhalb der ersten 10 Tage nach Fieberbeginn und Therapie mit i.v.-Gabe von Immunglobulinen kann die Prävalenz von Koronararterienaneurysmata und anderen Komplikationen auf etwa 4 % gesenkt werden [5]. Eine rasche Diagnosestellung ist somit ausschlaggebend für den Therapieerfolg und die Verbesserung der kurz- und langfristigen Prognose [3–5, 11]. Problematisch sind dabei das v. a. in Europa seltene Auftreten der Erkrankung, die weitgehend unspezifische klinische Präsentation und die Vielzahl an Differenzialdiagnosen. Die Diagnosestellung erfolgt nach definierten klinischen Kriterien (Tab. 1), wobei in den vergangenen Jahrzehnten wiederholt von verschiedenen Autoren aufgezeigt wurde, dass die Kriterien zu restriktiv sind und hieraus eine verzögerte Diagnosestellung und Therapieeinleitung resultieren [3, 5, 11]. In dem hier vorgestellten Fallbeispiel bestand als einziges Symptom wenige Tage lang anhaltendes Fieber, sodass die klinischen Diagnosekriterien bei Fehlen der in Tab. 1 aufgeführten Hauptsymptome nicht hinreichend erfüllt sind. Dennoch zeigten sich postmortal Veränderungen der Koronararterien, welche im gegenständlichen Fall zum raschen Todeseintritt geführt haben und in erster Linie typisch für das Kawasaki-Syndrom sind, wobei aufgrund der Überschneidungen auch eine infantile Polyarteriitis zu diskutieren ist. Es drängt sich die Frage auf, ob eine frühzeitige Therapieeinleitung den Krankheitsverlauf hätte verändern können. Da dies aus rechtsmedizinischer Sicht nicht mit der geforderten Sicherheit beweisbar sein wird, muss von einem schicksalhaften Verlauf einer endogenen Grunderkrankung ausgegangen werden.

Zusammenfassend ist somit der Leitbefund, der bei einer Obduktion an infantile Polyarteriitis und/oder ein Kawasaki-Syndrom denken lassen muss, das Vorliegen von Aneurysmata der Koronararterien, insbesondere bei Kindern, aber auch bei Erwachsenen im Sinne von Spätkomplikationen. Eine Mikroskopie belegt die lymphohistiozytäre Vaskulitis. Anamnestische Angaben, wie etwa das Vorliegen von Fieber, sind im Rahmen einer rechtsmedizinischen Obduktion keine validen Indikatoren für das Vorliegen einer Autoimmunvaskulitis.

## Fazit für die Praxis

- Das Kawasaki-Syndrom ist i. Allg. selten und tritt eher bei Kindern und jungen Erwachsenen als primär-endogene Autoimmunvaskulitis auf.
- Bei der Obduktion sind Aneurysmata der Koronararterien der zentrale makroskopische Leitbefund, der für ein Kawasaki-Syndrom sprechen kann.
- Die Mikroskopie beweist die lymphohistiozytäre Vaskulitis und kann helfen, andere Differenzialdiagnosen auszuschließen.

## References

[1] Barut K, Sahin S, Kasapcopur O (2016). Pediatric vasculitis. Curr Opin Rheumatol.

[2] Becker H, Höfler H, Urban C, Grubbauer H, Beitzke A (1981). Mukokutanes Lymphknotensyndrom in Österreich – Vier Fälle mit einem letalen Ausgang2. Teil: Pathomorphologische Befunde. Klin Padiatr.

[3] Boven K, De Graeff-Meeder ER, Spliet W, Kuis W (1992). Atypical kawasaki disease: an often missed diagnosis. Eur J Pediatr.

[4] Burns JC, Glodé MP (2004). Kawasaki syndrome. Lancet.

[5] Council on Cardiovascular Disease in the Young, Committee on Rheumatic Fever, Endocarditis, Kawasaki Disease, American Heart Association (2001). Diagnostic guidelines for Kawasaki disease. Circulation.

[6] Dettmeyer R, Amberg R, Varchmin-Schultheiß K, Madea B (1998). Sudden cardiac death due to atypical isolated coronary arteritis?. Forensic Sci Int.

[7] Fink W, Haidinger G (2007). Die Häufigkeit von Gesundheitsstörungen in 10 Jahren Allgemeinpraxis. Z. Allg..

[8] Kawasaki T, Kosaki F, Okawa S, Shigematsu I, Yanagawa H (1974). A new infantile acute febrile mucocutaneous lymph node syndrome (MLNS) prevailing in Japan. Pediatrics.

[9] Kawaski T (1967). Acute febrile mucocutaneous syndrome with lymphoid involvement with specific desquamation of the fingers and toes in children. Arerugi.

[10] Markwerth P, Bajanowski T, Tzimas I, Dettmeyer R (2020). Sudden cardiac death—update. Int J Legal Med.

[11] McCrindle BW, Rowley AH, Newburger JW, Burns JC, Bolger AF, Gewitz M, Baker AL, Jackson MA, Takahashi M, Shah PB, Kobayashi T, Wu MH, Saji Tsutomu T, Pahl E (2017). Diagnosis, treatment, and long-term management of Kawasaki disease: a scientific statement for health professionals from the American heart association. Circulation.

[12] Morand A, Urbina D, Fabre A (2020). COVID-19 and Kawasaki like disease: the known-known, the unknown-known and the unknown-unknown. Preprints.

[13] Niehues T (2013). The febrile child: diagnosis and treatment. Dtsch Arztebl Int.

[14] Smith AD (1977). Infantile polyarteritis and Kawasaki disease. Acta Paediatr.

[15] Takafuji H, Hosokawa S, Ogura R, Hiasa Y (2019). Combined bilateral giant coronary aneurysm and coronary fistula to coronary sinus. Cardiol J.

[16] Vural U, Kizilay M, Aglar AA (2019). Coronary Involvement in Behçet’s Disease: what are its Risks and Prognosis? (Rare Cases and Literature Review). Braz J Cardiovasc Surg.

